# Polymorphisms of the PPAR-*γ* (rs1801282) and Its Coactivator (rs8192673) Have a Minor Effect on Markers of Carotid Atherosclerosis in Patients with Type 2 Diabetes Mellitus

**DOI:** 10.1155/2016/4934251

**Published:** 2016-02-02

**Authors:** Aleš Pleskovič, Marija Šantl Letonja, Andreja Cokan Vujkovac, Jovana Nikolajević Starćević, Danijel Petrovič

**Affiliations:** ^1^Department of Cardiology, University Medical Centre Ljubljana, Zaloška 2, SI-1000 Ljubljana, Slovenia; ^2^Department of Radiology, General Hospital Murska Sobota, Ulica Dr. Vrbnjaka 6, SI-9000 Murska Sobota, Slovenia; ^3^Department of Internal Medicine, General Hospital Slovenj Gradec, Gosposvetska Cesta 1, SI-2380 Slovenj Gradec, Slovenia; ^4^Institute of Histology and Embryology, Faculty of Medicine, University in Ljubljana, Vrazov Trg 2, 1000 Ljubljana, Slovenia; ^5^Cardiology Outpatient Department, MC Medicor, 1000 Ljubljana, Slovenia

## Abstract

*Background*. The present study was designed to clarify whether common single nucleotide polymorphisms (SNPs) of the Peroxisome Proliferator-Activated Receptor-*γ* (PPAR-*γ*) gene (rs1801282) and the Peroxisome Proliferator-Activated Receptor-*γ* Coactivator-1 (PGC-1*α*) gene (rs8192673) are associated with markers of carotid and coronary atherosclerosis in Caucasians with type 2 diabetes mellitus (T2DM).* Patients and Methods*. 595 T2DM subjects and 200 control subjects were enrolled in the cross-sectional study. Markers of carotid atherosclerosis were assessed ultrasonographically. In 215 out of 595 subjects with T2DM, a coronary computed tomography angiography (CCTA) was performed for diagnostic purposes. Genotyping of either rs1801282 or rs8192673 was performed using KASPar assays.* Results*. In our study, we demonstrated an effect of the rs1801282 on markers of carotid atherosclerosis (presence of plaques) in Caucasians with T2DM in univariate and in multivariable linear regression analyses. Finally, we did not demonstrate any association between either rs1801282 or rs8192673 and markers of coronary atherosclerosis.* Conclusions*. In our study, we demonstrated a minor effect of the rs1801282 on markers of carotid atherosclerosis (presence of plaques) in Caucasians with T2DM. Moreover, we demonstrated a minor effect of the rs8192673 on CIMT progression in the 3.8-year follow-up in Caucasians with T2DM.

## 1. Introduction

Patients with diabetes mellitus have an increased risk of premature atherosclerosis [1, 2]. Type 2 diabetes mellitus (T2DM) causes more than a twofold increase in the incidence of myocardial infarction and coronary artery disease- (CAD-) related death [3].

The Peroxisome Proliferator-Activated Receptor-*γ* (PPAR-*γ*) and its coactivator, the Peroxisome Proliferator-Activated Receptor-*γ* Coactivator-1 (PGC-1*α*), are important molecules in atherogenesis because they are associated with metabolic risk factors, such as obesity and diabetes [4, 5]. PPAR-*γ* regulates insulin sensitivity by transcriptionally activating adipocyte-specific genes involved in insulin signaling, glucose uptake, fatty acid uptake, and lipid-storage [6]. Moreover, PPAR-*γ* plays an important role in adipogenesis and subcellular metabolism of arterial wall macrophage foam cells [6, 7]. Furthermore, the pharmacological PPAR-*γ* agonist thiazolidinedione drugs appear to be antiatherogenic at multiple levels, which include a generalized improvement of metabolism reduction of triglyceride accumulation, beneficial effects on vascular wall components (macrophages), and an improvement of the outcome of atherosclerotic disease [8–11].

Genetic polymorphisms of the PPAR-*γ* and PGC-1*α* genes have so far been reported to be associated with metabolic and cardiovascular end points [4, 5, 12–15]. A meta-analysis of 8 case-control studies and 2 family-based studies found that the PPARG A12 allele was associated with a reduced risk of type 2 diabetes [12]. The PPARG A12 allele was also associated with a reduced risk of myocardial infarction [13].

The aim of this study was to clarify whether common single nucleotide polymorphisms (SNPs) of the Peroxisome Proliferator-Activated Receptor-*γ* (PPAR-*γ*) gene (rs1801282) and the Peroxisome Proliferator-Activated Receptor-*γ* Coactivator-1 (PGC-1*α*) gene (rs8192673) are associated with markers of carotid atherosclerosis (carotid intima media thickness (CIMT), the number of affected segments of carotid arteries, and the sum of plaques thickness) in subjects with T2DM in the Caucasian population. The second aim of the study was to demonstrate an association between either rs1801282 or rs8192673 and the subclinical markers of CAD in the subset of patients with T2DM.

## 2. Methods

### 2.1. Patients

In this cross-sectional study 595 subjects with type 2 diabetes and 200 nondiabetic individuals were enrolled. The Slovene Medical Ethics Committee approved the study protocol. They were selected among patients admitted to the diabetes outpatient clinics of the general hospitals in Murska Sobota and Slovenj Gradec, Slovenia, and from the Cardiology Outpatient Department, MC Medicor, Ljubljana. Patients were classified as having T2DM according to the current report of the American Diabetes Association [16]. Patients were excluded if they had homozygous familial hypercholesterolaemia or a previous cardiovascular event such as myocardial infarction or a cerebral stroke. Clinical data, including smoking habits, duration and treatment of diabetes, arterial hypertension, hyperlipidemia, and consuming any other drugs were obtained from medical records and questionnaires. Patients were asked if they were smokers at the time of recruitment (current smoker).

Two experienced doctors blinded to the participants' diabetes status performed all ultrasound examinations. The CIMT, defined as the distance from the leading edge of the lumen-intima interface to the leading edge of the media-adventitia interface, was measured, as described previously [17]. Plaques were defined as a focal intima-media thickening and divided into 5 types according to their echogenic/echolucent characteristics, as described previously [17]. The interobserver reliability for carotid plaque characterization was found to be substantial (*κ* = 0.64, *p* < 0.001).

Control ultrasound examination was performed on 426 patients with diabetes and 137 healthy controls after 3.8 ± 0.5 years from the first examination. We used the annual CIMT progression rate, the increase in total plaque thickness, and the number of sites with plaques as well as the presence of unstable plaques as markers of carotid atherosclerosis progression.

In 215 out of 595 subjects with T2DM, a coronary computed tomography angiography (CCTA) was performed for diagnostic purposes. In 215 subjects with T2DM, coronary calcium score was measured and the presence of CAD was determined. Four regions (left main (LM), Left anterior descending (LAD) artery, left circumflex (LCX) artery, and right coronary artery (RCA)) were analyzed for the presence of CAD and more than 50% stenotic lesions were looked for in LM, LAD, LCX, RCA regions.

### 2.2. Biochemical Analyses

Blood samples for biochemical analyses, total cholesterol, triglyceride levels, high-density lipoprotein (HDL), low-density lipoprotein (LDL) cholesterol level, fasting blood glucose and glycated hemoglobin (HbA1c), hsCRP, and fibrinogen, were collected after a 12-hour fasting period. All the blood biochemical analyses were determined by using standard biochemical methods in the hospital's accredited lab.

### 2.3. Genotyping

The genomic DNA was extracted from 100 *μ*L of whole blood using a FlexiGene DNA isolation kit, in accordance with the recommended protocol (Qiagen GmbH, Hilden, Germany). Polymorphisms rs1801282 of the PPAR-*γ* gene and rs8192673 of the PGC-1*α* gene were determined with real-time PCR using StepOne*™* (48-well) Real-Time PCR Systems (Applied Biosystems, Foster City, CA, USA).

## 3. Statistical Analysis

Continuous variables were expressed as means ± standard deviations, when normally distributed, and as median (interquartile range) when asymmetrically distributed. Normality of the continuous variables was examined by the Kolmogorov-Smirnov test. Continuous clinical data were compared using an unpaired Student's *t*-test or analysis of variance (ANOVA) when normally distributed and the Mann-Whitney *U* test or the Kruskal-Wallis *H*-test when asymmetrically distributed. The Pearson *χ* ^2^ test was used to compare discrete variables and to test whether the genotypes distribution is in Hardy-Weinberg equilibrium. Pearson's correlation was performed to examine the association between independent variables. Due to the high correlation of systolic blood pressure with the diastolic blood pressure (*r* = 0.57, *p* < 0.001) they were not included together in the same statistical model. For the same reason the body mass index (BMI) was not included in the model together with the waist circumference (*r* = 0.45, *p* < 0.001).

Multivariable linear regression analysis was performed to determine the association of the tested polymorphisms with the CIMT/annual progression of CIMT and change in number of sites with plaque/total plaque thickness. To determine the association of the tested polymorphisms with the presence of atherosclerotic plaques on the carotid arteries or the presence of unstable plaques a multivariate logistic regression analysis was performed. All the regression models were adjusted for the presence of well established cardiovascular risk factors: age, gender, hypertension, systolic blood pressure, smoking, plasma levels of LDL and HDL cholesterol, triglycerides, HbA1c, and statin treatment. The results were presented as standardized *β* coefficients and *p* values for the linear regression and by odds ratios and 95% CIs for the logistic regression. A two-tailed *p* value less than 0.05 was considered statistically significant. A statistical analysis was performed using the SPSS program for Windows version 20 (SPSS Inc., Chicago, IL).

## 4. Results

Patients with T2DM had a greater waist circumference and higher fasting glucose and HbA1c levels compared to controls, whereas there were no differences in BMI or systolic and diastolic blood pressure between patients with T2DM and control subjects (Table 1). Patients with T2DM had lower total, HDL, and LDL cholesterol levels and a higher triglyceride level compared to controls (Table 1). Plasma level of inflammatory marker hsCRP was higher in patients with T2DM compared to controls (Table 1). Additionally, there were a higher percentage of men, statin therapy, and antihypertensive therapy and a lower percentage of smokers in the T2DM group compared to the control group (Table 1).

No statistically significant differences in the rs1801282 and rs8192673 genotype distribution frequencies were observed between T2DM patients and controls (Table 2). The rs1801282 genotype distributions in both patients with DM2 (*χ* ^2^ = 0.66; *p* = 0.42) and controls (*χ* ^2^ = 3.79; *p* = 0.05) were compatible with Hardy-Weinberg expectations. The rs8192673 genotype distributions in both patients with DM2 (*χ* ^2^ = 1.52; *p* = 0.22) and controls (*χ* ^2^ = 0.50; *p* = 0.48) were compatible with Hardy-Weinberg expectations (Table 2).

The comparison of atherosclerosis parameters was performed with regard to different genotypes of both polymorphisms (rs1801282, rs8192673) upon enrolment (Tables 3 and 4). In our study, we demonstrated an effect of the rs1801282 on the presence of plaques on subjects with T2DM by univariate and multivariable linear regression analysis (Tables 3 and 5), but we did not demonstrate any association between either the rs1801282 or the rs8192673 and other markers of carotid atherosclerosis CIMT, the sum of plaque thickness, the presence of unstable carotid plaques (Tables 3 and 4).

Finally, we did not demonstrate any association between either rs1801282 or rs8192673 and markers of coronary atherosclerosis obtained with CCTA (coronary calcium score, the number of coronary arteries with more than 50% stenosis and the presence of at least one vessel with more than 50% stenosis) in subjects with T2DM (Tables 3 and 4).

In our study, we demonstrated an effect of the rs8192673 on CIMT progression in the 3.8-year follow-up (Table 6). Using the multivariable linear regression analysis we demonstrated an effect of the rs1801282 on the presence of plaques in Caucasians with T2DM (Table 6).

## 5. Discussion

In the present study we tested the hypothesis that the rs1801282 of the PPAR-*γ* gene and the rs8192673 of the PGC-1*α* gene may be genetic markers of subclinical atherosclerosis of carotid and coronary arteries. In our study, we demonstrated an effect of the rs1801282 on markers of carotid atherosclerosis (presence of plaques) in Caucasians with T2DM in univariate and in multivariable linear regression analyses. The rs1801282 of the PPAR-*γ* gene was found to have a protective role against the development of atherosclerosis. Moreover, we demonstrated a minor effect of the rs8192673 on CIMT progression in Caucasians with T2DM in the 3.8-year follow-up.

In our study, we did not demonstrate any association between either the rs1801282 or the rs8192673 and CIMT, despite some previous reports on an association between the rs1801282 and CIMT [18–20]. In few populations (German population, Japanese population, and Canadian Oji-Cree Aborigines), the rs1801282 (Ala12 allele of the PPAR-*γ*) was reported to be associated with reduced CIMT [18–20]. Contrary to the lack of effect on CIMT, an effect of the rs8192673 on the CIMT progression rate and an effect of the rs1801282 on the presence of plaques in Caucasians with T2DM were demonstrated in univariate and in multivariable linear regression analyses. The rs1801282 of the PPAR-*γ* gene was found to have protective role against the development of atherosclerosis.

In the present study we pursued the hypothesis that either the rs1801282 of the PPAR-*γ* gene or the rs8192673 of the PGC-1*α* gene may be genetic markers of coronary atherosclerosis in subjects with T2DM. In our study, however, we did not demonstrate any association between either the rs1801282 or the rs8192673 and markers of coronary atherosclerosis obtained with CCTA (coronary calcium score, number of coronary arteries with more than 50% stenosis, and the presence of at least one vessel with more than 50% stenosis). Our findings are in accordance with the study of Nemoto and coworkers on 91 subjects with T2DM, in which they failed to demonstrate the effect of the variability in the PPAR-*γ* gene on the coronary calcium score [21]. However, in several studies the effect of polymorphisms of PPAR*γ*2/PGC-1*α* genes on CAD/MI risk was reported [1, 13, 15, 22–24]. In their case-control study, Galgani and coworkers demonstrated that homozygosity for the Ala allele at codon 12 of the PPAR*γ*2 (rs1801282) gene was associated with a reduced risk of CAD [22]. Similarly, Ridker and coworkers reported in a prospective study that the rs1801282 of the PPAR-*γ* (A12 allele) was associated with a 25% reduction in myocardial infarction risk [13]. Ding and coworkers, however, failed to demonstrate a significant effect of the rs1801282 of the PPAR-*γ* on CAD risk in their meta-analysis (74 studies with 52,998 subjects included) [23]. Cresci and coworkers reported a variant (rs1503298) in a single PPAR pathway gene (i.e., TLL1) that was associated with the extent of CAD in patients with T2DM and CAD [15].

Potential mechanisms of the effect of the variants of both genes (PPAR-*γ*, PGC-1*α*) may be speculated to affect serum/tissue levels of both proteins, other risk factors (i.e., obesity and obesity indexes) or other effects (i.e., lipid status).

In our recently published study, we demonstrated that the rs8192673 of the PGC-1*α* gene and the rs1801282 of the PPAR-*γ* gene have been associated with waist circumference in subjects with T2DM [4]. Huang and coworkers demonstrated the effect of the rs1801282 of the PPAR-*γ* gene in the meta-analysis (74 studies with 52,998 subjects) on lipid parameters [25]. They reported that subjects (male) with the AlaAla genotype had lower blood TG than subjects with ProPro genotype in Caucasians [25].

Strength of our study is the community-based sample and the detailed phenotypic characterization of the subjects with regard to ultrasonically determined carotid atherosclerosis, as well as having data of a rather large sample of subjects with T2DM. A limitation is the use of cross-sectional data in the analysis, restricting the possibility of causal inferences from our data and allowing for bias. An additional limitation is that while we assume that the effect of the PPAR-*γ*/PGC-1*α* gene variants on plaque is due to their influence on serum/tissue levels of the respective enzymes, we do not have any direct measure to be able to investigate this.

## 6. Conclusions

To conclude, in our study we demonstrated a minor effect of the rs1801282 on markers of carotid atherosclerosis (presence of plaques) in Caucasians with T2DM. Moreover, we demonstrated a minor effect of the rs8192673 on CIMT progression in the 3.8-year follow-up. Our findings suggest that the tested polymorphisms in the PPAR-*γ*/PGC-1*α* genes play a minor role in the development of subclinical atherosclerosis in subjects with T2DM.

## Figures and Tables

**Table 1:** Baseline clinical and biochemical characteristics of diabetic patients and controls.

	Diabetic patients *n* = 595	Controls *n* = 200	*p*
Age (years)	61.38 ± 9.65	60.07 ± 9.18	0.07
Male gender (%)	338 (56.8)	92 (46.0)	**0.008**
DM duration (years)	11.25 ± 7.88	—	—
Smoking prevalence (%)	53 (8.91)	34 (17.0)	**0.002**
Statin therapy (%)	375 (63.0)	62 (31.0)	<0.001
Antihypertensive agents (%)	499 (83.8)	58 (29%)	<0.001
Waist circumference (cm)	108.65 ± 12.88	93.31 ± 13.18	**<0.001**
BMI (kg/m^2^)	30.96 ± 4.74	27.90 ± 4.42	0.16
Systolic blood pressure (mm Hg)	146.98 ± 19.98	143.3 ± 16.6	0.86
Diastolic blood pressure (mm Hg)	85.75 ± 11.62	84.7 ± 11.6	0.19
Fasting glucose (mmol/L)	8.04 ± 2.57	5.27 ± 0.87	**<0.001**
HbA1c (%)	7.89 ± 3.56	4.79 ± 0.29	**<0.001**
Total cholesterol (mmol/L)	4.70 ± 1.19	5.36 ± 1.08	**<0.001**
HDL cholesterol (mmol/L)	1.19 ± 0.35	1.43 ± 0.37	**<0.001**
LDL cholesterol (mmol/L)	2.63 ± 0.94	3.24 ± 0.98	**<0.001**
Triglycerides (mmol/L)	1.9 (1.2–2.7)	1.3 (0.9–1.9)	**<0.001**
hsCRP (mg/L)	2.2 (1.0–4.3)	1.3 (0.8–2.7)	**<0.001**
CIMT (*μ*m)	958 ± 194	890 ± 212	**0.007**

DM: diabetes mellitus; hsCRP: high sensitivity C-reactive protein.

**Table 2:** Genotype distribution and allele frequencies of the polymorphisms rs1801282 and rs8192673 in patients with T2DM and in control subjects.

	Subjects with T2DM *n* = 595	Control subjects *n* = 200	*p*
rs1801282			
CC genotype	422 (70.9)	137 (68.5)	0.27
GC genotype	155 (26.1)	52 (26.0)	
GG genotype	18 (3.0)	11 (5.5)	
C allele	999 (83.9)	326 (81.5)	0.26
G allele	191 (16.1)	74 (18.5)	
rs8192673			
TT genotype	309 (52.0)	92 (46.0)	0.28
TC genotype	231 (38.8)	84 (42.0)	
CC genotype	55 (9.2)	24 (12.0)	
T allele	849 (71.4)	268 (67.0)	0.10
C allele	341 (28.6)	132 (33.0)	

**Table 3:** Ultrasonographic markers of carotid atherosclerosis due to rs1801282 genotypes in patients with T2DM at the time of recruitment.

Pro12AlaPPAR	CC	GC + GG	*p*
CIMT (*μ*m)	1006 ± 210	1026 ± 209	0.39
Number of sites with plaque	2.56 ± 1.57	2.36 ± 1.82	0.31
Total plaque thickness (mm)	7.98 ± 4.47	7.65 ± 4.64	0.58
Presence of plaques			
+	365 (86.5)	133 (76.9)	**0.005**
−	57 (13.5)	40 (23.1)	
Presence of unstable plaques			
+	213 (58.4)	74 (55.6)	0.61
−	152 (41.6)	59 (44.4)	
Coronary calcium score^*∗*^	250 ± 315	269 ± 367	0.1
Number of coronary arteries with more than 50% stenosis	0.7 ± 0.9	0.9 ± 1.2	0.4
The presence of at least 1 vessel with more than 50% stenosis^*∗*^	24 (38.9%)	63 (41.1%)	0.2

^*∗*^Coronary computed tomography angiography (CCTA) was performed for diagnostic purposes in 215 out of 595 subjects with T2DM.

**Table 4:** Ultrasonographic markers of carotid atherosclerosis due to rs8192673 genotypes in patients with T2DM at the time of recruitment.

	TT	TC	CC	*p*
CIMT (*μ*m)	1007 ± 224	1012 ± 191	1012 ± 217	0.94
Number of sites with plaque	2.36 ± 1.60	2.69 ± 1.66	2.72 ± 1.67	0.13
Total plaque thickness (mm)	7.54 ± 4.54	8.06 ± 4.82	8.31 ± 4.35	0.35
Presence of plaques				
+	257 (83.2)	194 (84.0)	47 (85.5)	0.90
−	52 (16.8)	37 (16.0)	8 (14.5)	
Presence of unstable plaques				
+	149 (58.0)	108 (55.7)	29 (61.7)	0.73
−	108 (42.0)	86 (44.3)	18 (38.3)	
Coronary calcium score^*∗*^	181 ± 170	344 ± 376	200 ± 304	0.2
Number of coronary arteries with more than 50% stenosis^*∗*^	0.7 ± 1.1	0.9 ± 1.1	0.6 ± 1.3	0.8
The presence of at least 1 vessel with more than 50% stenosis^*∗*^	7 (31.8%)	33 (39.3%)	48 (44.0%)	0.3

^*∗*^Coronary computed tomography angiography (CCTA) was performed for diagnostic purposes in 215 out of 595 subjects with T2DM.

**Table 5:** Association of the rs1801282 genotypes with the presence of plaques and presence of unstable plaques in patients with T2DM at the time of recruitment.

	Presence of plaque		Presence of unstable plaque	
	OR (95% CI)	*p*	OR (95% CI)	*p*
rs1801282				
Hypertension (0 = no; 1 = yes)	1.71 (0.93–2.58)	**0.04**	1.25 (0.88–2.64)	0.97
Systolic blood pressure (mm Hg)	1.07 (0.92–1.007)	0.17	1.11 (0.86–1.44)	0.32
LDL cholesterol (mmol/L)	1.21 (0.78–1.89)	0.40	1.08 (0.75–1.56)	0.67
HDL cholesterol (mmol/L)	0.18 (0.05–0.63)	**0.008**	0.30 (0.08–1.13)	0.08
Triglycerides (mmol/L)	1.28 (0.63–1.03)	0.09	1.09 (0.66–1.37)	0.34
Hba1c (%)	1.14 (0.64–1.54)	0.28	1.22 (0.74–1.92)	0.42
GC + GG^*∗*^	0.79 (0.48–1.14)	**0.04**	0.83 (0.34–1.91)	0.65
rs8192673				
Hypertension (0 = no; 1 = yes)	1.35 (1.13–1.93)	**0.04**	1.15 (0.75–2.77)	0.79
Systolic blood pressure (mm Hg)	1.08 (0.96–1.34)	0.16	1.02 (0.97–1.25)	0.31
LDL cholesterol (mmol/L)	1.22 (0.78–1.89)	0.29	1.07 (0.74–1.54)	0.73
HDL cholesterol (mmol/L)	0.19 (0.05–0.72)	0.54	0.29 (0.08–1.05)	0.06
Triglycerides (mmol/L)	1.34 (0.63–1.90)	0.09	1.19 (0.66–1.59)	0.34
Hba1c (%)	1.16 (0.65–2.06)	0.32	1.11 (0.86–1.44)	0.42
TC^*∗∗*^	0.97 (0.56–1.38)	0.48	1.16 (0.59–2.70)	0.55
CC^*∗∗*^	1.08 (0.38–1.34)	0.63	1.43 (0.43–4.73)	0.56

All the models were adjusted for age, gender, smoking, and statin treatment.

^*∗*^Reference group were homozygotes for allele C. ^*∗∗*^Reference group were homozygotes for allele T.

**Table 6:** Association of the rs1801282 genotypes with ultrasonographic markers of carotid atherosclerosis progression in patients with T2DM.

	CIMT progression rate		Δ Number of sites with plaque		Δ Total plaque thickness	
	*β*	*p*	*β*	*p*	*β*	*p*
rs1801282						
Hypertension (0 = no; 1 = yes)	0.013	0.92	0.020	0.90	0.069	0.26
Systolic blood pressure (mm Hg)	0.022	0.52	0.052	0.69	0.037	0.82
LDL cholesterol (mmol/L)	0.057	0.69	0.051	0.71	0.073	0.49
HDL cholesterol (mmol/L)	−0.211	0.19	−0.230	0.14	−0.189	0.37
Triglycerides (mmol/L)	0.249	0.13	0.343	0.78	0.359	0.44
Hba1c (%)	1.151	0.29	1.097	0.83	1.176	0.41
GC + GG^*∗*^	0.818	0.93	0.728	0.18	0.684	0.16
rs8192673						
Hypertension (0 = no; 1 = yes)	0.140	0.37	0.062	0.71	0.069	0.29
Systolic blood pressure (mm Hg)	0.186	0.25	0.046	0.88	0.075	0.35
LDL cholesterol (mmol/L)	0.172	0.59	0.143	0.75	0.446	0.35
HDL cholesterol (mmol/L)	−0.203	0.18	−0.232	0.08	−0.192	0.34
Triglycerides (mmol/L)	0.168	0.28	0.117	0.43	0.127	0.21
Hba1c (%)	0.146	0.27	0.143	0.26	0.228	0.16
TC^*∗∗*^	0.068	0.63	−0.066	0.64	0.328	0.32
CC^*∗∗*^	0.349	**0.01**	−0.115	0.11	0.681	0.06

All the models were adjusted for age, gender, smoking, statin treatment and baseline value of dependent variable.

^*∗*^Reference group were homozygotes for the allele C; ^*∗∗*^Reference group were homozygotes for the allele T.
